# Rhino-Orbital Cerebral Mucormycosis: A Fatal Evolution

**DOI:** 10.7759/cureus.37837

**Published:** 2023-04-19

**Authors:** Said Benlamkaddem, Ghita Zdaik, Djoudline Doughmi, Ahmed Bennis, Fouad Chraibi, Mohamed Adnane Berdai, Meriem Abdellaoui, Idriss Benatiya Andaloussi, Mustapha Harandou

**Affiliations:** 1 Maternal and Pediatric Critical Care Unit, Hassan II University Hospital, Fez, MAR; 2 Faculty of Medicine and Pharmacy, Sidi Mohamed Ben Abdellah University, Fez, MAR; 3 Department of Ophthalmology, Hassan II University Hospital, Fez, MAR

**Keywords:** rhino-orbital mucormycosis, rhino-orbital cerebral mucormycosis, multi-organ dysfunction, liposomal amphotericin b, icu-acquired immunosuppression, fungal infection, mucormycosis

## Abstract

Rhino-orbital cerebral mucormycosis is a rare and serious fungal infection caused by fungi of the *Mucorales* order, most commonly by the species *Rhizopus oryzae*. It occurs generally in an immunocompromised host, and the contamination of healthy subjects remains exceptional. The clinical presentation is not specific. The diagnosis of rhino-orbital cerebral mucormycosis is difficult based on a range of clinical, microbiological, and radiological arguments. Imaging studies may include CT/MRI of the orbit, brain, and sinuses and show signs of aggressiveness, intracranial complications, and evolution under treatment. The standard treatment is antifungal therapy and necrosectomy. We report a case of a 30-year-old patient admitted to intensive care for the management of postpartum hemorrhage complicating severe preeclampsia who presented with rhinocerebral mucormycosis with left orbital extension. Adequate therapeutic management in the intensive care unit was provided; however, the patient died within seven days of septic shock with multiorgan failure. The mortality is determined by the correction of risk factors, the timing of initiation of the antifungal therapy, and surgical debridement.

## Introduction

Mucormycosis is a rare, fast-spreading, opportunistic mycosis with a poor prognosis [1]. It is caused by filamentous fungi of the *Mucorales* type, which are ubiquitous saprophytes of the soil and many plant substrates. Mucormycosis can become pathogenic in very specific contexts, especially in cases of severe immunosuppression [2,3].

Its usual clinical presentation is acute sinusitis progressing to ophthalmic and central nervous system involvement [2,3]. The definitive diagnosis of mucormycosis is mycological and/or histological. Computed tomography is essential in the diagnostic process and the extension assessment. Its prognosis is poor, depending essentially on the precocity of the diagnosis [2-4].

## Case presentation

A 30-year-old female, gravida 4 para 2, was admitted to the intensive care unit for the management of severe postpartum hemorrhage due to uterine rupture after a homebirth of stillborn twins in the context of severe preeclampsia. At her first examination, she was tachycardic at 140 bpm, hypotensive at 90/60 mmHg, oligoanuric, and confused. She also had jaundice and proteinuria. Laboratory tests showed hemolytic anemia (anemia with increased lactate dehydrogenase (LDH) and low haptoglobin), thrombocytopenia, a low prothrombin time (PT), acute kidney injury with high blood urea nitrogen (BUN) and serum creatinine, liver cytolysis (increased aspartate aminotransferase (ASAT) and alanine aminotransferase (ALAT)), hyperbilirubinemia, and hypoalbuminemia (Table 1).

**Table 1:** Laboratory findings at admission. ASAT: aspartate aminotransferase; ALAT: alanine aminotransferase; LDH: lactate dehydrogenase.

Parameters	Admission	Normal range
Hemoglobin (g/dL)	10	11.5-15
Platelets (x10^3^/µL)	33	150-450
Prothrombin time (PT, %)	35	>70
Haptoglobin (g/l)	0.04	0.3-2
ASAT (UI/L)	1690	0-35
ALAT (IU/L)	1079	0-35
Total bilirubin (mg/L)	235	<12
LDH (IU/L)	3300	0-240
Blood urea nitrogen (BUN, g/L)	0.8	0.1-0.4
Serum creatinine (mg/L)	30	5-13
Albumin (g/L)	23	35-52

This clinical picture was suggestive of multiorgan failure (renal and liver failure, hematological dysfunction) due to the combined effects of hemorrhagic shock and severe preeclampsia with HELLP (hemolysis, elevated liver enzymes, and low platelet) syndrome. The management was then based on hemodynamic stabilization with fluid resuscitation, and norepinephrine after invasive monitoring with transpulmonary thermodilution EV1000 (Edwards Lifesciences, Irvine, USA), hysterectomy under general anesthesia, transfusion with red packed cells (RPC), platelets, and fresh frozen plasma (FFP), venovenous hemofiltration, and magnesium sulfate (4 grams (gm) loading dose over 20 minutes, then 1 gm/hour for 24 hours). We added corticosteroids (hydrocortisone 200 mg/day) in the presence of persistent hypotension despite increasing norepinephrine doses (ranging between 0.1 and 0.8 micrograms (mcg)/kg/min) with low systemic vascular resistance (vasoplegia).

**Figure 1:**
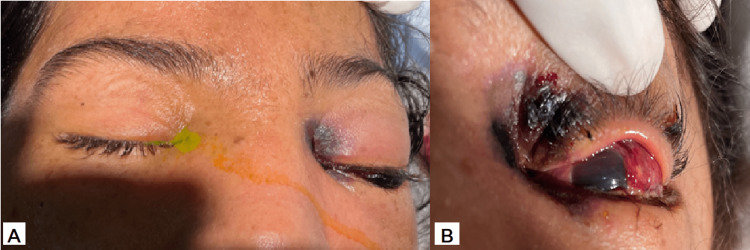
Image of the left eye showing (A) inflammatory palpebral edema and (B) skin necrosis proximal to the internal canthus, proptosis, and necrotic conjunctival chemosis.

On day nine, while all organ dysfunctions were being stabilized, we observed a palpebral necrosis with edema in the left eye. The ophthalmological examination of the right eye found inflammatory palpebral edema, a conjunctival chemosis with abundant purulent secretions, a clear cornea, a negative fluorescein test, a clear lens, correct ocular tone, and a normal fundus. Examination of the left eye found inflammatory palpebral edema, skin necrosis proximal to the internal canthus, proptosis, and necrotic conjunctival chemosis with a very edematous cornea that did not allow us to appreciate the details of the anterior chamber (Figure 1).

**Figure 2:**
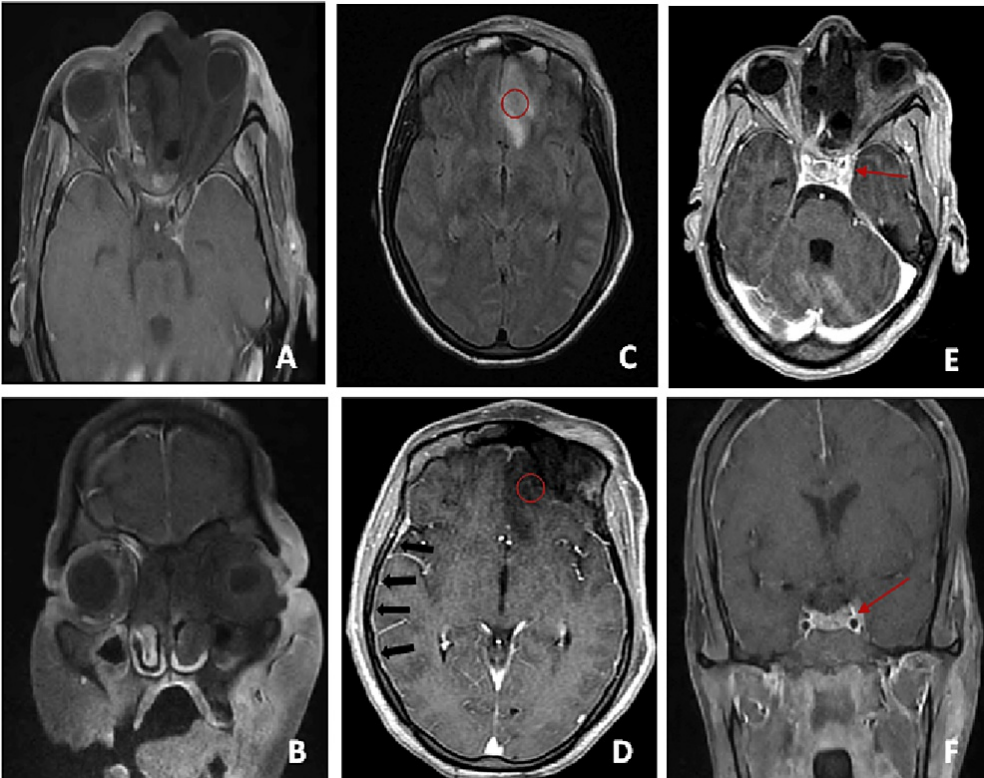
Enhanced T1W axial (A) and coronal (B) MRI sequences showing the black turbinate: lack of enhancement on postcontrast related to frontal, ethmoidal, sphenoidal, and maxillary sinuses necrosis with left orbital extension complicated by grade II left proptosis. Axial brain FLAIR MRI (C) showing a frontal lobe hyperintensity with no T1W contrast-enhancement (D) (circle) associated with meningeal enhancement (black arrows) due to meningoencephalitis. Enhanced three-dimensional T1W axial (E) and coronal (F) sequences showing enlargement of the left cavernous sinus with lack of T1W contrast-enhancement related to sinus thrombosis (red arrows). T1W: T1-weighted; FLAIR: fluid-attenuated inversion recovery.

Examination of the nasal cavities revealed black eschar lesions on the nasal mucosa with epistaxis on the left side, justifying the urgent prescription of intravenous amphotericin B after performing a bacteriological and mycological sample. Magnetic resonance imaging of the paranasal sinuses (fat-saturated T2-weighted (T2W) and three-dimensional (3D) T1-weighted (T1W) contrast-enhanced sequences) showed frontal, ethmoidal, sphenoidal, and maxillary sinuses necrosis, which appear hypointense on T1W images with a lack of enhancement on postcontrast with left orbital extension complicated by grade II left proptosis (Figures 2A, 2B). The brain MRI using a standard protocol (turbo spin echo (TSE) T1W and T2W imaging, fluid attenuation inversion recovery (FLAIR), diffusion-weighted imaging (DWI), and contrast administration) revealed a frontal lobe T2W and FLAIR hyperintensity with no 3D T1W contrast-enhancement associated to meningeal enhancement due to meningoencephalitis (Figures 2C, 2D). There was also an enlargement of the left cavernous sinus with a lack of 3D T1W contrast-enhancement related to sinus thrombosis (Figures 2E, 2F).

Surgical debridement with skin and conjunctival necrosectomy was performed associated with intravitreal amphotericin B injection (dilution of 10 mcg/0.1 ml). Intravenous liposomal amphotericin B was urgently started at a dose of 10 mg/kg/day. Mycological examination confirmed the presence of *Mucorales*-type fungi.

The evolution was unfavorable, and the patient died seven days later in the context of septic shock with multiorgan failure.

## Discussion

First recognized by J.E. Gregory in 1943, mucormycosis is an acute, opportunistic, and progressive fungal disease. It is caused by *Mucorales* family fungal infections. The main agents of human infection are *Rhizopus*, *Mucor*, *and Absidia* [5]. Contamination usually occurs by inhalation of the spores, explaining the preferred naso-sinus and pulmonary locations, and more rarely by ingestion or transcutaneous inoculation. These fungi only become pathogenic in a particular environment, especially immunosuppression, and diabetic ketoacidosis [2,3]. Mucores have a high vascular tropism, causing thrombosis and tissue necrosis.

Rhino-orbito-cerebral mucormycosis is the most common clinical form, particularly in poorly balanced diabetic patients (60-80% of cases) [6]. Other risk factors have been described, such as blood diseases, neutropenia, metabolic acidosis, prolonged treatment with corticosteroids, transplantation of solid organs or bone marrow, chemotherapy, and renal failure. The contamination of healthy subjects remains exceptional [2,4,7].

In this case, the immune system was compromised by a confluence of multiple aggressions such as hemorrhagic shock, preeclampsia with HELLP syndrome, and multiorgan dysfunction syndrome (MODS). All these conditions are characterized by the overactivation of inflammatory cells with a massive liberation of cytokines. At the same time, an immunosuppressive state occurs to offset the hyperinflammation by exerting negative feedback through anti-inflammatory cytokine liberation (interleukin 10, cortisol, prostaglandin E2, etc.) [8]. This ICU-acquired immunosuppression exposes critically ill patients to bacterial hospital-acquired infections, fungal infections, and viral reactivations. Additionally, corticosteroids, used to increase the sensitivity to vasopressors and to improve systemic vascular resistance, can also impact the immune system and reduce its capacity [2].

The diagnosis of rhino-orbito-cerebral mucormycosis is difficult. It should be based on a conjunction of clinical, microbiological, and radiological arguments. The clinical presentation is not specific, especially at the onset of the disease, which may mislead early diagnosis [2,9]. Pulmonary, cutaneous, and disseminated forms are less frequent. They are more common in patients with hematological malignancies or immunosuppressive treatment [2,4,5].

Ophthalmologically, it is manifested as proptosis; ophthalmoplegia is sometimes associated with ptosis [10]. Ocular paralysis is secondary to paralysis of the cranial nerves or direct damage to the oculomotor muscles. Proptosis secondary to tissue infiltration and/or cavernous sinus thrombosis is reported in 64-83% of cases [10]. The extension of the infection to the orbital apex can cause optic neuritis, a source of blindness (65-80%). Endogenous mucor endophthalmitis is rarely observed [10]. The decrease in visual acuity is most often due to thrombosis of the ophthalmic vein, which is considered a specific sign of mucormycosis [11].

Although computed tomography is not specific, it helps to assess the extension, look for signs of aggressiveness, identify intracranial complications, and follow the evolution under treatment [6]. It demonstrates sinus involvement in almost all cases; the most affected sinuses are the sphenoid, ethmoid, and then maxillary [2,12]. Cavernous sinus thrombosis is a sign of poor prognosis. The diagnosis of mucormycosis is confirmed by the demonstration on direct mycological examination and/or pathological examination of irregular, non-septate mycelial filaments with right-angled ramifications [2,3], an important inflammatory response, vascular invasions by the mycelial filaments, thromboses, necroses, and hemorrhages of the surrounding tissues. Histological examination may present certain diagnostic difficulties due to the fragmentation of fungal elements. The interest of culture is to identify the species. A positive culture alone cannot confirm the diagnosis of mucormycosis except in the presence of the characteristic clinical signs of this disease [2]. *Rhizopus oryzae* is the most frequently encountered species in the literature [3,11]. The identification of species has an epidemiological and especially therapeutic interest, the sensitivity of *Mucorales* to antifungals being variable [13,14]. However, in some cases, the culture may remain negative, hence the interest in molecular biology methods. Serology has no place in the diagnosis of mucormycosis [15,16].

The treatment of mucormycosis is based on the association of an antifungal with the surgical debridement of necrotic tissue. Liposomal amphotericin B is used as first-line monotherapy. Daily doses ranged from 1 mg/kg per day to 10 mg/kg/day, with a substantial risk of renal toxicity that is mostly reversible. Doses of 10 mg/kg/day are indicated in cases of central nervous system (CNS) involvement, as in our case. In the absence of CNS involvement, amphotericin B lipid complex (5 mg/kg/day) has been used successfully. Amphotericin B deoxycholate has been the drug of choice for decades. It is associated with a high risk of toxicity that limits its use to situations when there is no other antifungal therapy available [2]. The combination of an antifungal with surgical debridement is essential because of the poor diffusion of antifungals in necrotic tissues [3]. Correction of any other risk factor, such as diabetes control, is necessary. Adjuvant treatment may help to limit local extension and increase the chance of recovery. It includes functional endoscopic sinus surgery, retrobulbar and intravitreal injection of amphotericin B, and hyperbaric oxygenation because of its fungistatic role and its role in the neovascularization of ischemic territories [17-19]. In addition to the absence of surgical treatment and the delay in diagnosis, other factors of poor prognosis have been identified in the literature, such as neurological involvement, the presence of facial necrosis, and hyperglycemia [3,6].

## Conclusions

Mucormycosis is a rare condition with high mortality. Usually, very rapid growth to fulminant disease is the cause. A clinical picture with an eschar is often tell-tale. A multidisciplinary team approach is opted along with a surgeon (ENT and ophthalmologist) for urgent debridement, functional endoscopic sinus surgery, and retrobulbar injection of amphotericin B, infectious disease specialist, and critical care medicine specialist.
